# Emerging Microbiological and Sensor-Based Approaches for Biofilm Detection in Meat and Poultry Processing Environments

**DOI:** 10.3390/foods15173015

**Published:** 2026-08-27

**Authors:** Corliss A. O’Bryan, Bailey Stalford, Tomi Obe, Philip G. Crandall

**Affiliations:** 1 Department of Food Science, University of Arkansas-Fayetteville, Fayetteville, AR 72701, USA; stalford@uark.edu; 2 Department of Poultry Science, University of Arkansas-Fayetteville, Fayetteville, AR 72701, USA; toobe@uark.edu

**Keywords:** biofilm detection, meat processing, poultry processing, advanced microscopy, molecular methods, Extracellular polymeric substances, biosensors, real-time monitoring

## Abstract

Biofilms remain a major challenge in meat and poultry processing because conventional sanitation verification methods provide only indirect evidence of attached microbial communities. Emerging microbiological and sensor-based technologies offer new opportunities to improve biofilm detection by providing information on biofilm structure, cellular membrane integrity, composition, and spatial distribution. This review evaluates advanced imaging techniques, molecular assays, extracellular polymeric substance (EPS)-focused analyses, and real-time sensor platforms for their potential to strengthen risk-based biofilm monitoring in meat and poultry processing environments. Confocal and epifluorescence microscopy, scanning electron microscopy, optical coherence tomography, and in situ fluorescence imaging provide detailed visualization of biofilm architecture and viability, supporting validation of routine monitoring methods and assessment of sanitation practices. Quantitative PCR, digital PCR, amplicon sequencing, and metagenomics characterize biofilm communities, identify persistent microorganisms, and evaluate sanitation effectiveness, while EPS analyses of polysaccharides, proteins, extracellular DNA, and lipids indicate biofilm maturity and resilience. Electrochemical impedance, quartz crystal microbalance, surface acoustic wave sensors, and microfluidic platforms show promise for near-real-time detection of attached biomass. Collectively, these technologies provide a framework for more targeted, data-driven biofilm surveillance that can improve sanitation verification and reduce pathogen persistence in meat and poultry processing facilities.

## 1. Introduction

Biofilms in meat and poultry processing plants are increasingly recognized as persistent, system-level hazards that connect equipment design, sanitation practices, and regulatory expectations, all of which must work together to effectively control pathogens [1,2,3]. In the United States, chicken is the most widely consumed meat, and poultry products remain a major source of *Salmonella* and *Listeria monocytogenes* infections, with repeated outbreaks and recalls linked to persistent contamination in processing environments [4,5]. Recent surveillance and outbreak investigations in major U.S. poultry-producing regions continue to demonstrate the importance of controlling *Salmonella*, *Campylobacter*, and *Listeria* contamination throughout poultry processing [6,7]. Table 1 documents several recent outbreaks. At the same time, numerous studies have shown that cold, wet processing environments, including evisceration equipment, chillers, and other continuously wet surfaces, can support persistent biofilms that serve as reservoirs for recurrent contamination despite routine sanitation, highlighting the need for more sensitive and targeted biofilm detection strategies [7,8].

While classical and plant-ready tools such as visual inspections, ATP and protein swabs, culture-based environmental monitoring, and commercial biofilm revealers provide the practical backbone of current detection programs, each method offers only a partial and often indirect view of where biofilms are established on complex, cold, and wet processing surfaces [2,15,16]. However, these methods typically provide only indirect evidence of attached microbial communities [17], may fail to detect localized contamination within difficult-to-access microenvironments, are poorly suited for surveying large or complex equipment geometries [18], and generally cannot distinguish transient organic residues from mature, EPS-embedded biofilms on cold, wet processing surfaces [19,20]. Likewise, ATP bioluminescence and protein-based hygiene assays primarily indicate residual organic material rather than biofilm architecture or viability and therefore cannot reliably determine whether persistent biofilms are present within hard-to-clean niches that contribute to recurring contamination in poultry processing facilities [20].

In parallel with these established tools, a rapidly expanding body of research has investigated emerging microbiological and sensor-based approaches for biofilm detection in food processing environments. Advanced imaging techniques, including confocal laser scanning microscopy (CLSM), epifluorescence microscopy, scanning electron microscopy (SEM), optical coherence tomography (OCT) [21], and fluorescence imaging, together with molecular methods such as quantitative PCR (qPCR), digital PCR (dPCR), amplicon sequencing, and metagenomics, provide detailed information on biofilm architecture, microbial community composition, and spatial organization. In addition, EPS-targeted analytical methods and real-time biosensor platforms, including electrochemical impedance spectroscopy (EIS) [22], quartz crystal microbalance (QCM) [23], surface acoustic wave (SAW) sensors, and microfluidic devices [24], have demonstrated considerable promise for detecting attached biomass and monitoring biofilm development. Although many of these technologies remain at the laboratory or pilot scale, increasing efforts are focused on adapting them for routine monitoring in meat and poultry processing environments [7,19,25].

Several recent reviews have addressed biofilm detection in the food industry broadly or have focused on specific technology classes, including EPS characterization, microbiome profiling, molecular diagnostics, and biosensors [19,26,27]. However, these reviews generally (i) consider multiple food sectors collectively without emphasizing the distinctive environmental conditions, sanitation challenges, and regulatory context of meat and poultry processing, (ii) focus primarily on either microbiological characterization or engineering aspects of detection technologies rather than integrating both perspectives, and (iii) provide relatively little practical guidance for incorporating emerging detection methods into routine sanitation verification programs that currently rely on ATP bioluminescence, protein assays, and culture-based testing. Consequently, meat and poultry processors have limited consolidated guidance regarding which advanced biofilm detection technologies are suitable for routine monitoring, which are appropriate for rapid screening, which require confirmatory testing before a biofilm is considered established, which are best reserved for validation and root-cause investigations, and which remain primarily research tools. This review addresses that gap by evaluating emerging microbiological and sensor-based approaches for biofilm detection specifically in meat and poultry processing environments, with emphasis on poultry facilities. We focus on how advanced imaging, molecular assays, EPS-targeted chemistries, and real-time sensor platforms can complement, refine, or partially replace classical plant-ready tools in a tiered monitoring framework that supports risk-based sanitation decisions and compliance with FSIS guidance. For each tool class, we highlight evidence from poultry meat, poultry-processing environments, and other poultry-related products where available, and we explicitly identify applications that are currently conceptual for poultry but supported by data from other meat or non-food systems (Table 2).

Operationally, the proposed framework separates rapid screening from confirmation and from investigative or validation methods. Tier 1 methods are intended to rapidly identify sites that warrant attention but generally do not establish the presence, identity, or viability of a biofilm-forming microorganism. Tier 2 methods provide orthogonal microbiological confirmation, using culture, targeted molecular assays, or both, depending on whether the objective is recovery of viable organisms, detection of a specific target, or quantitative assessment. Tier 3 methods are primarily used to investigate biofilm structure, community composition, persistence, sanitation response, or performance of emerging detection technologies. Importantly, signals generated by fluorescence, ATP, EIS, QCM, or SAW should not be interpreted as definitive evidence of biofilm without an appropriate confirmatory measurement.

Literature search and classification approach. Because this is a narrative review, we performed a focused literature search rather than a formal systematic review. Articles were identified primarily through Web of Science, Scopus, PubMed, and Google Scholar using combinations of terms such as “biofilm,” “meat processing,” “poultry processing,” “chicken,” “sanitation,” “sensor,” “impedance,” “qPCR,” “digital PCR,” “amplicon sequencing,” “metagenomics,” “EPS,” and “optical coherence tomography,” with emphasis on studies published from approximately 2000 through early 2026. We prioritized studies conducted in meat and poultry processing environments or plant-like laboratory systems but included selected clinical, environmental, and engineering studies when they provided mechanistic or conceptual insights relevant to meat and poultry plants. Throughout, we classify tools as “plant-ready” when at least one documented commercial deployment or broadly marketed industrial product is available for meat or poultry processing and as “emerging” when evidence is confined to laboratory, pilot-scale, or conceptual demonstrations.

## 2. Advanced Microscopy and Imaging

Advanced microscopy and imaging methods allow the determination of 3D biofilm architecture and spatial heterogeneity, and (with differential stains) the viability and matrix organization, in ways that swabs and plate counts cannot. In meat and poultry processing plants and pilot facilities, these tools can explain *why* niches persist (surface finish, cold/wet conditions, organic soils such as meat/poultry exudate) and validate simpler routine monitoring methods rather than replacing them [21,28,29]. Rather than replacing routine monitoring methods, advanced imaging is best used to validate ATP, culture-based, and other sanitation verification tools, investigate persistent contamination events, and identify harborage niches on food-contact surfaces under realistic processing conditions. Much of the detailed understanding of biofilm structure and EPS organization originates from laboratory investigations, with increasing validation in meat and poultry processing environments [30,31]. Table 3 summarizes the following information.

### 2.1. Confocal Laser Scanning Microscopy, Epifluorescence, and Scanning Electron Microscopy

#### 2.1.1. Confocal Laser Scanning Microscopy (CLSM)

Confocal laser scanning microscopy (CLSM) is widely used in the laboratory to examine harvested biofilms because it can scan multiple optical sections through the biofilm and reconstruct 3D structures, which is critical when thickness and stratification are important. In meat and poultry research, CLSM has been applied primarily to biofilms grown on stainless-steel coupons, processing materials, and plant-derived samples under laboratory or pilot-scale conditions, where it has been used to evaluate biofilm development and sanitation effectiveness. A key advantage is that CLSM is compatible with fluorescent probes that report on physiology and chemistry (not just morphology), enabling a paired structural visualization and “functional” readouts such as viability, extracellular DNA, and polysaccharide-rich regions [28,29].

To assess cell-envelope integrity, CLSM is commonly used with fluorescent nucleic-acid stains such as SYTO 9 and propidium iodide (PI). SYTO 9 can stain cells with intact or compromised membranes, whereas PI preferentially enters cells with compromised membranes. The resulting fluorescence patterns therefore indicate membrane integrity and spatial heterogeneity within the biofilm rather than a definitive live/dead classification. Membrane-compromised cells may be injured but potentially recoverable, whereas membrane-intact cells may include dormant or viable-but-non-culturable (VBNC) populations. Accordingly, SYTO 9/PI imaging should be interpreted as a complementary indicator of cellular membrane status rather than as a stand-alone measure of viability [29].

CLSM also supports EPS visualization by pairing cell stains with matrix-targeted fluorescent probes (e.g., lectins for polysaccharides and other EPS components), enabling spatial mapping of EPS distribution relative to cells and biofilm voids. Although stain choice strongly affects interpretation, this capability allows investigators to determine whether a current intervention reduced biomass by stripping away the EPS matrix, killing cells without removing the entire scaffold, or merely dispersing surface layers while leaving an EPS-rich basal film on the food-contact surface [32]. Much of the mechanistic understanding of these structural responses originates from laboratory biofilm studies, whereas applications in commercial meat and poultry processing have primarily focused on validating sanitation interventions and investigating persistent contamination sites.

#### 2.1.2. Epifluorescence Microscopy

Epifluorescence microscopy is less expensive and faster than CLSM, making it well suited for rapid screening of laboratory-inoculated coupons (e.g., stainless steel) or for scraped material from processing plants after staining an existing biofilm to confirm attachment and general viability patterns. In meat and poultry biofilm studies, conventional fluorescence or epifluorescence microscopy is commonly used to verify surface-associated cells, estimate two-dimensional surface coverage, and obtain preliminary live/dead staining patterns. However, unlike CLSM, it does not provide optical sectioning or robust depth-resolved measurements [15,29]. Thus, images of thick or particulate-associated biofilms, such as those formed in the presence of meat or poultry exudates, should be interpreted cautiously, because projected two-dimensional signals can obscure spatial heterogeneity in cell viability and matrix distribution [15,29,33].

#### 2.1.3. Scanning Electron Microscopy (SEM)

Scanning electron microscopy (SEM) provides high-resolution surface morphology that is well-suited for showing attachment phenotypes, microcolony footprints, and interactions with surface topography, such as scratches and pits on stainless steel processing surfaces. In meat and poultry research, SEM has been used primarily to characterize biofilms on stainless steel coupons, equipment materials, and processing-line samples collected for laboratory analysis. Important limitations include the dehydration and fixation steps required during sample preparation, which can alter the appearance of the EPS matrix, and conventional SEM’s inability to directly assess cell viability. Thus, it is most informative when paired with fluorescence/CLSM or culture-based measures in a multimodal design [28,30].

Under plant-like laboratory conditions, stainless steel coupons are commonly used as surrogates for processing equipment to evaluate biofilm development and sanitation effectiveness. While CLSM or epifluorescence microscopy can quantify surface coverage and estimate biofilm mass from stained areas, SEM can reveal how cells spread across scratch marks and settle into small surface defects that can become difficult-to-clean niches after repeated use. Combining these methods is especially useful under plant-like conditions (low temperatures, periodic moisture, and complex food residues), provides a more complete understanding of the heterogeneous biofilm structures, patches, streamers, and protected microcolonies that are often overlooked by bulk sampling methods [21,29].

CLSM can also be combined with matrix-targeted fluorescent probes, such as lectins, to localize EPS components relative to bacterial cells and identify heterogeneous matrix-rich regions. These measurements can help characterize how sanitation treatments affect biofilm structure and provide mechanistic information that complements culture-based and molecular measurements. When paired with viability indicators and quantitative image analysis, these measurements can help distinguish treatment-associated reductions in matrix signal, loss of cellular viability with retained matrix-associated signal and decreases in superficial biofilm biovolume. Such mechanistic inferences are derived primarily from controlled laboratory biofilm models, but CLSM and related approaches have also been used to examine biofilms from meat-processing environments and to assess cleaning-and-disinfection performance [34,35,36].

In meat and poultry systems, food residues and biofilm-matrix constituents can interfere with fluorescence-based interpretation. Therefore, imaging should include matrix-matched unstained controls, single-stain controls, and soil-only controls; acquisition settings should be fixed across comparison groups, with spectral overlap, autofluorescence, and detector saturation assessed before quantitative interpretation of EPS-associated signals [15,37].

### 2.2. In Situ and Non-Destructive Imaging

The key limitation of conventional microscopy for plant-relevant applications is that it requires skilled laboratory personnel and often relies on destructive sampling (coupons, cutouts, or scraping) and/or extensive sample preparation, which can alter the very structures we want to understand. In contrast, in situ and non-destructive modalities aim to image biofilms on intact surfaces, as close as possible to the “as-found” state, and to do so quickly enough to support operational decisions, additional sanitation, or repeated measurements over time [30].

#### Optical Coherence Tomography (OCT)

Optical coherence tomography (OCT) is a valuable noninvasive, label-free method for imaging mesoscopic biofilm structure. It can visualize internal architecture and quantify thickness or other structural parameters in situ without destructive sectioning, enabling repeated measurements of the same sample during biofilm development or exposure to flow, shear, and cleaning-related treatments [21,38,39].

In meat- and poultry-processing research, OCT has been discussed or applied primarily in laboratory-scale and model-system contexts rather than as a routine online plant-monitoring method. Such model studies, including work with flow channels, stainless steel or other process-relevant surfaces, and food-processing water hoses, demonstrate OCT’s potential to track biofilm growth, deformation, detachment, and residual structure over time [7,40,41].

Accordingly, OCT can provide useful dynamic information on biofilm response to simulated cleaning or hydrodynamic stress, although treatment-associated structural loss should be complemented with microbiological or chemical measurements when conclusions concern cell viability, species composition, or matrix composition [21,38].

### 2.3. In Situ Fluorescence Systems and 3D Imaging

In situ fluorescence approaches, including fixed and portable imaging systems, can bridge the gap between laboratory microscopy and commercial meat and poultry processing by allowing rapid scanning for fluorescent signatures after staining or under intrinsic fluorescence/reflectance regimes. Although these systems do not provide the optical sectioning or three-dimensional resolution of CLSM, they can be used operationally to identify areas requiring additional sampling, targeted sanitation, or confirmatory laboratory analyses [30].

A current example of this direction is Bactiscan, a macro-scale, reagent-less fluorescence-based device for detecting microbial contamination that has been evaluated on stainless steel surfaces and tested under food processing conditions, including commercial chicken processing [42]. These studies suggest that Bactiscan can rapidly identify areas of elevated microbial contamination and help direct subsequent ATP testing, microbiological sampling, or microscopy. However, current evidence supports its use as a complementary screening tool rather than a replacement for established sanitation verification methods [42].

Importantly, fluorescence-based imaging systems should be regarded as indicator technologies rather than definitive biofilm detection methods. While they can rapidly identify potential hotspots, including drains, conveyor frameworks, hollow rollers, gasket interfaces, and other difficult-to-clean locations, they cannot reliably distinguish mature biofilms from fluorescent food residues, meat and poultry exudates, or residual cleaning chemicals. Consequently, positive findings should be confirmed using culture-based methods, microscopy, or other biofilm-specific analytical techniques before corrective actions are based solely on fluorescence signals [42].

### 2.4. Strengths, Limitations, and Relevance to Meat and Poultry Processing

The principal strength of advanced imaging methods is the depth of information they provide. CLSM combines high-resolution three-dimensional visualization with compositional and viability information through fluorescent staining, whereas OCT offers non-destructive, time-resolved monitoring of biofilm structure that conventional endpoint assays cannot provide. Most applications in meat and poultry research have been conducted under laboratory or plant-like conditions using stainless steel coupons, pilot-scale systems, or equipment samples, where these techniques have proven valuable for investigating why sanitation interventions succeed or fail, comparing cleaning chemistries under realistic soil conditions, and determining whether apparently clean surfaces remain colonized by persistent biofilms [30].

Despite these advantages, several factors currently limit routine implementation in commercial meat and poultry processing plants. Instrumentation costs, specialized operator training, and sample preparation requirements, particularly for SEM and fluorescence-based methods requiring careful staining and controls, restrict their practical use for routine sanitation verification [21,28]. In addition, imaging techniques evaluate relatively small surface areas, making them susceptible to sampling bias unless study designs incorporate appropriate replication and strategic sampling. Because biofilms on commercial processing equipment are often highly heterogeneous and localized within difficult-to-clean niches, imaging methods are generally best suited for validation studies, root-cause investigations, and sanitation optimization rather than routine plant-wide surveillance [43]. Taken together, advanced imaging methods are best viewed as validation and investigative tools rather than routine sanitation monitoring methods. Their greatest value in meat and poultry processing lies in confirming biofilm presence, identifying persistent harborage sites, evaluating sanitation interventions, and providing mechanistic insight that can improve plant hygiene programs.

**Table 3:** Characteristics, evidence base, technology readiness, and recommended applications of advanced microscopy and imaging methods for biofilm detection in meat and poultry processing environments.

Method	Principle/Information Provided	Typical Deployment	Evidence Base	Technology Readiness	Advantages	Limitations	Primary Role in Meat & Poultry Processing
Confocal laser scanning microscopy (CLSM) [27,28]	Three-dimensional optical sectioning with fluorescent probes; biofilm thickness, membrane integrity, EPS distribution	Laboratory; stainless steel coupons; pilot-scale studies; equipment samples	Plant-like laboratory systems; limited commercial validation	Validation tool	Excellent 3D visualization; spatial assessment of membrane integrity; EPS localization	Expensive instrumentation; specialized expertise; destructive sampling; limited field of view	Validate sanitation procedures; investigate persistent contamination; characterize EPS; root-cause analysis
Epifluorescence microscopy [25,36]	Two-dimensional fluorescence imaging of stained biofilms	Laboratory coupons; plant scrapings; equipment samples	Plant-like laboratory systems	Validation tool	Rapid; relatively inexpensive; useful screening method	No depth information; limited EPS characterization	Rapid confirmation of biofilm presence; preliminary screening before advanced analyses
Scanning electron microscopy (SEM) [21,44]	High-resolution imaging of surface morphology and attachment patterns	Laboratory analysis of coupons and removed equipment components	Plant-like laboratory systems	Research/Validation	Exceptional surface detail; identifies scratches, pits, and harborage sites	Dehydration alters EPS; no viability information; labor intensive	Characterize harborage sites; evaluate equipment design; investigate sanitation failures
Optical coherence tomography (OCT) [24,31]	Non-destructive imaging of hydrated biofilms; thickness and structural dynamics	Laboratory flow cells; pilot-scale systems	Laboratory and pilot-scale systems	Emerging	Real-time monitoring; repeated measurements; tracks detachment and regrowth	Structural information only; requires complementary methods for viability and EPS	Research on cleaning dynamics; evaluation of sanitation strategies; longitudinal biofilm studies
In situ fluorescence imaging systems (e.g., Bactiscan) [35,45]	Portable or fixed fluorescence imaging of intact equipment surfaces	Commercial processing plants; pilot facilities	Commercial validation with continued technology development	Commercial screening tool	Rapid screening of large areas; identifies hotspots; operationally practical	Cannot distinguish biofilm from food residues or cleaning chemicals; confirmatory testing required	Direct ATP, culture, or microscopy sampling; prioritize sanitation efforts

## 3. Molecular and Sequencing-Based Approaches

Modern molecular methods, from targeted PCR assays to community-level sequencing, are now central to detecting, quantifying, and understanding biofilms in food processing environments. Each tool offers distinct strengths, trade-offs, and practical considerations for routine monitoring versus deeper investigations [40,46,47,48,49,50,51]. Table 4 summarizes the following information.

### 3.1. Quantitative PCR and Digital PCR

Quantitative PCR (qPCR) remains the most widely used molecular method for targeted detection and quantification of microorganisms in food safety and environmental monitoring because it measures fluorescence during DNA amplification and converts the cycle threshold (Ct) value to target copy number using a standard curve [49]. In meat and poultry processing, qPCR has been applied to detect and quantify foodborne pathogens, characterize biofilm-associated microorganisms, and assess sanitation effectiveness on environmental and equipment samples [52]. Compared with qPCR, dPCR offers greater precision and sensitivity for detecting low-abundance targets and is particularly advantageous when complex food matrices or PCR inhibitors limit qPCR performance. Although qPCR is well established in food microbiology laboratories, dPCR remains an emerging technology for routine meat and poultry biofilm investigations because of its higher instrumentation costs and more limited commercial adoption [44,49,53].

#### qPCR for Biofilm and Gene-Targeted Assays

Quantitative PCR (qPCR) can quantify targeted microbial populations or genes, but interpretation depends on the target, extraction efficiency, gene copy number, matrix composition, and assay design. DNA-based qPCR detects target DNA regardless of whether the originating cell is viable; extracellular DNA and DNA released from dead cells can therefore persist and contribute to positive signals. Target copy-number variation among bacterial taxa also means that gene copies should not automatically be interpreted as cell numbers, particularly when multicopy targets such as 16S rRNA genes are used. RNA-based RT-qPCR can provide information more closely associated with physiological activity because RNA generally turns over more rapidly than DNA; however, RNA stability varies among targets and organisms, and RNA extraction and preservation can introduce substantial technical variation. Consequently, RNA-based assays should be interpreted as indicators of transcriptional activity or physiological state rather than definitive measures of viable or metabolically active cell numbers [54,55,56].

These limitations are particularly relevant to biofilms, in which viable, injured, dormant, and viable-but-non-culturable (VBNC) cells may coexist with dead cells and extracellular nucleic acids. Standard DNA-qPCR cannot distinguish between these populations. Propidium monoazide (PMA)- or PMAxx-qPCR can reduce amplification from DNA associated with membrane-compromised cells and extracellular DNA, providing a membrane-integrity-based estimate of viable-cell abundance. PMA/PMAxx approaches, however, require optimization for the organism, target, matrix, and killing treatment and do not directly measure metabolic activity; membrane-intact VBNC cells may remain detectable [57,58,59].

In meat and poultry processing environments, qPCR has been applied to detect foodborne pathogens, quantify antimicrobial resistance genes, and measure universal bacterial markers in environmental biofilm samples, enabling comparisons among sampling sites, sanitation treatments, and processing conditions. Thus, qPCR and RT-qPCR are best viewed as complementary molecular tools that provide target-specific information on microbial abundance and, where appropriately designed, physiological state rather than as stand-alone measures of viability.

### 3.2. Amplicon Sequencing and Metagenomics

Sequencing approaches add an ecological context. Amplicon sequencing (e.g., bacterial 16S rRNA; fungal ITS) rapidly profiles “who is there,” whereas shotgun metagenomics sequences all DNA, enabling species/strain resolution and functional gene insights [40,46,47,48,50,51].

#### 3.2.1. Characterizing Biofilm Communities in Meat and Poultry Processing

Amplicon surveys of equipment, drains, and hoses consistently reveal diverse microbial communities, often dominated by psychrotrophic spoilage organisms, including *Pseudomonas*, Enterobacteriaceae, and *Paenibacillus*. In dairy and similar facilities, 16S profiles frequently showed overlaps between product-contact surfaces and surrounding non-contact areas, supporting a facility-level resident microbiome that links community structure to quality/safety outcomes. Cold-tolerant spore formers can persist despite standard sanitation practices [40,46,47,48,50,51,60].

#### 3.2.2. Monitoring Sanitation Effectiveness and Persistence

Multiple surveys show that a relatively stable resident microbiome can endure for months or even years, especially in wet, hard-to-clean areas. Common members include psychrotrophic Gram-negative rods (e.g., *Pseudomonas*, *Acinetobacter*, *Comamonas*, *Flavobacterium*, *Aeromonas*), lactic acid bacteria, and spore-formers (e.g., *Bacillus*, *Paenibacillus*). Amplicon datasets identify core taxa that recur across sites/time, while shotgun metagenomics can resolve strain-level patterns tied to persistent *Salmonella* embedded in mixed biofilms [40,46,47,48,50,51,60,61]. Collecting and analyzing biofilms at this level can facilitate root cause analysis and significantly improve pathogen control.

Pairing pre- and post-sanitation sampling with 16S profiling can quantify cleaning efficacy. In some facilities, genera such as *Paenibacillus*, *Viridibacillus*, and *Lysinibacillus* persist on product-contact surfaces even after cleaning, highlighting the resilience of these spore-formers. Studies comparing conventional versus enzymatic cleaning show that the overall spoilage organism fraction may remain similar, yet the composition within that group can shift substantially. Institutional kitchen metagenomics likewise points to taxa that persist despite routine cleaning and sanitation practices, suggesting environmental reservoirs rather than daily reintroduction [46,48,50,51].

Biofilms are rarely present as single-species systems. Environmental bacteria often enhance pathogen persistence: in vitro, genera such as *Pseudomonas*, *Comamonas*, *Acinetobacter*, *Flavobacterium*, *Janthinobacterium*, and *Aeromonas* can markedly increase *Salmonella* biofilm biomass when growing symbiotically. Network analyses of 16S data can pinpoint interacting core taxa that repeatedly co-occur at specific sites or zones, flagging candidate risk consortia [40,47,61,62,63].

Field and lab evidence support the presence of recurrent assemblages linked to biofilm mass, sanitation survival, or contamination. For example, repeated detection of *Pseudomonas*-dominated consortia alongside *Staphylococcus aureus* on food-contact surfaces suggests that these communities promote pathogen persistence and should be targeted as a combined unit rather than on a species-by-species basis. Mixed-species consortia can produce more biomass or tolerate more sanitizers than their strongest single-member signals. Tracking these consortia at critical sites (e.g., belts, drains, hoses) can guide interventions such as enzymatic cleaning, alternative chemistries, or mechanical modifications to disrupt community structure [40,47,48,50,51,64].

Sequence surveys help identify indicator organisms whose presence signals probable harborage of biofilm or co-occurrence with pathogens. Useful indicators tend to (1) persist pre/post cleaning, (2) sit strongly connected to pathogen nodes in co-occurrence networks, or (3) be enriched in biofilm-positive samples (e.g., hoses with OCT-visible biofilms). In practice, environmental genera such as *Pseudomonas*, *Acinetobacter*, *Comamonas*, and certain Enterobacteriaceae often serve this role in meat and poultry processing plants. Once identified, they can be tracked with targeted qPCR or rapid tools to monitor sanitation performance and trigger focused interventions at problem sites [40,46,47,48,50,51].

Collectively, sequencing studies demonstrate that biofilm persistence is often driven by stable multispecies communities rather than individual organisms. Identifying recurrent taxa, microbial interaction networks, and indicator organisms can improve root-cause analysis and enable targeted sanitation strategies focused on persistent harborage sites in meat and poultry processing environments.

#### 3.2.3. Shotgun Metagenomics

Shotgun metagenomics goes beyond taxonomy to functional analyses: it can detect biofilm-associated genes, sanitizer tolerance, stress responses, and virulence determinants, while also distinguishing closely related species or strains. In food plant biofilms, these data often reveal multispecies communities in which spoilage organisms and pathogens co-occur with environmental taxa across equipment, drains, and water infrastructure. Such signatures help link specific taxa or gene combinations to post-cleaning persistence, harborage niches, and the traceback of final-product contamination [40,47,50,51,65].

Although shotgun metagenomics remains primarily a research and root-cause investigation tool because of its cost, data complexity, and longer turnaround times, it provides information that cannot be obtained with targeted PCR assays alone. By simultaneously characterizing microbial community composition and functional potential, shotgun metagenomics can identify persistent reservoirs, track strain-level transmission within processing facilities, and guide interventions aimed at disrupting biofilm communities rather than targeting individual microorganisms. As sequencing costs continue to decline and bioinformatics pipelines become more accessible, shotgun metagenomics is expected to play an increasingly important role in validating sanitation programs and investigating persistent contamination events in meat and poultry processing facilities.

### 3.3. Practical Considerations for Molecular Monitoring

Turning sequencing and PCR results into actionable decisions requires careful attention to turnaround time, cost, metadata, and, crucially, how the results map back to specific equipment for remediation. In routine labs, qPCR typically delivers same-day to 1–2-day results; dPCR reporting time frame is similar or slightly longer due to the partitioning steps. In routine laboratories, qPCR typically delivers same-day to 1–2-day results, while dPCR reporting is similar or slightly longer because of the partitioning workflow. In routine laboratories, qPCR typically delivers same-day to 1–2-day results, while dPCR reporting is similar or slightly longer because of the partitioning workflow. Amplicon sequencing typically requires approximately 1–2 weeks, although end-to-end turnaround can be longer depending on extraction, library preparation, sequencing, batching, and bioinformatic analysis. Shotgun metagenomics generally requires several days to several weeks, depending on workflow and sequencing platform. dPCR involves higher instrument/consumable costs due to partitioning platforms but can reduce reliance on standard curves when absolute quantification or improved precision is essential. Amplicon sequencing is generally more cost-effective than shotgun sequencing for large sample numbers (small genomic region, heavy multiplexing), whereas shotgun sequencing carries higher library/sequencing and analysis costs, even as commercial pricing trends downward on higher volumes [66,67,68,69].

Environmental/equipment swabs often yield ultra-low biomass, increasing susceptibility to reagent contaminants and requiring strict controls and quantitative checks (e.g., parallel qPCR to verify biomass). Amplicon and shotgun data are inherently compositional; integrating them with absolute counts from qPCR/dPCR helps distinguish true increases from proportional shifts. Cross-run or cross-platform batch effects (primer choice, sequencer, and pipeline differences) also complicate longitudinal comparisons and require standardized methods to maintain trend fidelity [67,68,69,70,71].

**Table 4:** Characteristics, evidence base, technology readiness, and recommended applications of molecular methods for biofilm detection in meat and poultry processing environments.

Method	Information Provided	Typical Deployment	Evidence Base	Technology Readiness	Advantages	Limitations	Primary Role in Meat & Poultry Processing	Typical Turnaround
qPCR [72]	Detection and relative quantification of targeted microorganisms or genes	Food microbiology laboratories; environmental swabs	Commercial meat and poultry studies	Routine analytical tool	Rapid; sensitive; relatively inexpensive; established workflows	Requires target-specific primers; relative quantification; PCR inhibitors	Pathogen detection; AMR gene monitoring; sanitation verification	Same day to 24 h
Digital PCR (dPCR) [73,74]	Absolute quantification of DNA targets	Specialized laboratories	Commercial meat and poultry studies; laboratory validation	Validation tool	High sensitivity; absolute quantification; resistant to inhibitors	Higher instrumentation cost; lower throughput	Confirmatory testing; low-abundance pathogens; assay validation	1–2 days
Amplicon sequencing (16S rRNA/ITS) [37,47,51]	Microbial community composition and diversity	Research laboratories; environmental surveys	Commercial meat and poultry studies; plant-like laboratory systems	Emerging	Identifies resident microbiota; detects community shifts; relatively economical compared with shotgun sequencing	Limited functional information; generally resolves taxa to genus level; longer turnaround time	Characterize resident microbiota; identify persistent communities; evaluate sanitation effects	Approx. 1–2 weeks
Shotgun metagenomics [38,42]	Community composition plus functional genes, strain-level resolution, AMR, virulence, and stress-response genes	Research laboratories; root-cause investigations	Plant-like laboratory systems; limited commercial investigations	Research/Emerging	Comprehensive characterization; strain tracking; functional profiling; traceback capability	High cost; complex bioinformatics; longer turnaround; large data sets	Root-cause investigations; characterize persistent biofilms; identify functional traits associated with persistence	Several days to several weeks

* Evidence Base (for all tables).

• Commercial meat and poultry studies—Evaluated or validated using samples, surfaces, equipment, or process streams collected directly from operating commercial meat or poultry processing facilities.
• Plant-like laboratory systems—Evaluated using stainless-steel coupons, pilot-scale systems, flow cells, or simulated processing conditions relevant to meat and poultry.
• Laboratory proof-of-concept—Demonstrated under controlled laboratory conditions without direct food-processing validation.
• Conceptual adaptation—Supported by evidence from non-food or engineering systems but requiring validation in meat and poultry processing.

## 4. EPS-Focused Analyses

### 4.1. Quantitative EPS Assays

Extracellular polymeric substances (EPS) are key structural components of biofilms and typically include polysaccharides, proteins, extracellular DNA (eDNA), and lipids. In meat and poultry processing environments, EPS measurements must be interpreted within a complex food matrix because residual soils may already contain proteins, lipids, carbohydrates, and DNA that can overlap with assay targets and contribute background signal. Detergents, sanitizers, and rinse chemistries may further affect extraction efficiency or interfere with colorimetric, fluorescent, or enzymatic assays used to quantify EPS components [75,76,77,78]. EPS composition is highly dynamic and influenced by microbial strains, nutrient regime, shear, and age, making time-resolved compositional analysis capable of revealing trajectories from early adhesion through maturation to dispersal. For example, studies on environmental and pathogenic biofilms have shown progressive accumulation of carbohydrates in EPS and changes in protein and nucleic acid signatures over days of incubation, with strong correlations with increased biovolume and thickness [79,80,81].

Accordingly, EPS-based methods should be paired with appropriate controls, including soil blanks, reagent blanks, and recovery tests using representative processing soils. Where possible, results should also be normalized to surface area, total biomass, ATP, or another relevant denominator to improve comparability across surfaces and treatments. These steps are especially important when evaluating biofilms on food contact surfaces where residue load and sanitizer exposure can vary considerably.

The relationship between EPS composition and biofilm maturity should also be interpreted cautiously. Although increased polysaccharide, protein, or extracellular DNA content has been associated with greater biofilm persistence in some systems, these patterns are strongly influenced by strain, surface type, temperature, nutrient conditions, and cleaning history. In mixed-species biofilms typical of meat and poultry plants, EPS composition may therefore vary substantially across sites and production conditions. For this reason, EPS measurements are best considered complementary indicators of matrix development and potential resilience rather than stand-alone proof of mature biofilm formation.

#### 4.1.1. Quantitative Polysaccharide Assays

Polysaccharides in EPS extracts are usually quantified using colorimetric total carbohydrate assays, such as the phenol–sulfuric acid method or the anthrone assay, typically read in microplates and calibrated against glucose or other relevant standards [82]. These methods respond to a broad range of hexoses, pentoses, and uronic acids, providing a bulk estimate of the carbohydrate-rich EPS fraction from scrapings or swabs, albeit without structural specificity [75,76,78]. In some time-course studies, an increase in the total carbohydrate content of EPS has occurred as biofilms developed and became denser, or less porous; however, such relationships are context-dependent and should be interpreted as associations rather than universal indicators of maturity [79,80,81].

#### 4.1.2. Quantitative Protein Assays

EPS proteins are usually measured using microplate-adapted protein assays such as the Lowry, bicinchoninic acid (BCA), or micro-BCA methods, often after dialysis or precipitation to remove interfering detergents or chelators [78]. Nitrogen-based approaches (e.g., elemental analysis or the Kjeldahl method) may also be used to estimate the protein contribution to EPS when sufficient material can be recovered from heavily fouled surfaces [76,78,79]. In general, protein-rich EPS fractions have been associated with cohesion and mechanical stability in some biofilms, particularly under nutrient conditions that favor proteinaceous matrix development; however, these patterns vary with organism, medium, surface type, and environmental history and should not be generalized across systems without validation [78,79].

#### 4.1.3. Quantitative Extracellular DNA Assays

Extracellular DNA (eDNA) is commonly quantified using fluorescent intercalating dyes such as Pico Green or similar double-stranded DNA-selective reagents, which can be applied in 96- or 384-well plate formats for high-sensitivity detection of nanogram- to picogram amounts of DNA in EPS extracts. These assays provide a specific readout of double-stranded DNA after removal of intact cells (e.g., via centrifugation and filtration) and can be normalized to biomass or to the sampled surface area [76,78]. eDNA contributes to cell–cell adhesion and matrix organization, particularly during early biofilm development, and may continue to serve as a scaffold in more mature biofilms [76,77,80,83]. Nevertheless, eDNA abundance should be interpreted cautiously because signal intensity can be affected by cell lysis, sample handling, and the presence of intracellular DNA released during extraction.

#### 4.1.4. Quantitative Lipid Assays

Lipids and lipoproteins in EPS can be quantified by extracting the organic fraction (e.g., chloroform–methanol extraction) and performing gravimetric determination, colorimetric assays for total lipids, or chromatographic detection (e.g., HPLC, GC–MS) when higher resolution is required. Although lipids are measured less frequently than polysaccharides or proteins, they may contribute to hydrophobicity, barrier properties, and resistance to desiccation or antimicrobial penetration [75,76,78]. In some biofilm systems, lipid-rich fractions have been linked to enhanced surface conditioning and to cell aggregation; however, the relevance of these patterns to food-contact biofilms should be interpreted cautiously because lipid measurements may also be influenced by residual food material and other matrix components [75,76,78].

#### 4.1.5. Relating EPS Composition to Maturity and Robustness

Quantitative EPS assays for polysaccharides, proteins, eDNA, and lipids provide complementary information about biofilm maturity when applied to time-structured swabs or scrapings. In some cases, early-stage biofilms contain proportionally more eDNA and adhesion-associated proteins, whereas later stages show increased carbohydrate accumulation and more pronounced matrix structuring [77,79,80,81,83]; however, these patterns are strongly influenced by strain, surface type, nutrient availability, temperature, and cleaning history, and they should not be treated as universal markers of maturity. Nutrient conditions can also alter both total EPS yield and its internal composition, thereby affecting matrix cohesion and stress tolerance. Nutrient conditions can also alter both total EPS yield and internal composition, thereby affecting matrix cohesion and stress tolerance. Accordingly, EPS composition should be viewed as a context-dependent indicator of matrix development and potential robustness rather than as a stand-alone measure of biofilm age [78,79,84].

#### 4.1.6. Methodological Considerations and Limitations

Interpreting EPS assays from swabs or scrapings requires careful control of extraction efficiency, normalization, and matrix effects. In meat and poultry processing environments, residual soils may contain proteins, lipids, carbohydrates, and DNA that overlap with EPS analytes and may interfere with assay performance. Detergents, sanitizers, and rinse chemistries may further alter extraction efficiency or affect colorimetric, fluorescent, or enzymatic readouts [76,78]. For this reason, EPS-based methods should be paired with appropriate controls, including soil blanks, reagent blanks, and recovery tests using representative processing soils, and results should be normalized where possible to surface area, total biomass, ATP, or another relevant denominator. Moreover, bulk biochemical assays collapse heterogeneous EPS chemistries into single quantitative values, obscuring spatially localized features such as patches of eDNA–polysaccharide fibers or protein-rich microdomains. Combining quantitative EPS measurements from surface samples with imaging or spectroscopic modalities that resolve micro-scale distribution (e.g., CLSM with fluorescent lectins, Raman mapping) provides a more complete picture of how matrix composition underpins biofilm maturity and resilience in real processing environments [78,80,84].

## 5. Biosensors and Real-Time Platforms

Electrochemical impedance spectroscopy (EIS), quartz crystal microbalance (QCM), and surface acoustic wave (SAW) sensors all detect attached biomass by transducing biofilm-induced changes at a solid–liquid interface into electrical or acoustic signals, and each has been adapted or at least demonstrated in formats relevant to meat and poultry processing environments [45,85,86,87,88]. Table 5 summarizes the following information.

### 5.1. Impedance/EIS: Principles and Biofilm Sensing

Electrochemical impedance spectroscopy (EIS) measures the ease with which electrons can move between an electrode and a liquid. Scientists explain this behavior using a simple “electrical circuit” model that represents factors such as the liquid’s resistance, how charge builds up at the surface, how readily reactions occur, and, sometimes, how ions slowly move through the system [87,89].

When bacteria attach to microelectrodes and begin forming a biofilm, they alter the electrode’s electrical behavior. Cells and their EPS replace ions and water at the surface, altering surface capacitance, slowing or redirecting charge transfer, and changing how electrical signals move through the interface. As a result, biofilm growth can produce measurable changes in impedance magnitude and phase, but the frequency that provides the greatest sensitivity depends on electrode geometry, measurement configuration, organism, and surrounding medium. For example, a recent microfabricated EIS study using Pseudomonas aeruginosa PAO1 with interdigitated electrodes identified 1.26 kHz as an optimized measurement frequency in 1:10 tryptic soy broth (TSB), whereas 200 kHz was used in a 5% oil–water emulsion because the optimal response differed with the medium [87,89].

Microfabricated interdigitated electrodes incorporated into flow cells have demonstrated real-time tracking of biofilm growth and removal under controlled laboratory conditions. In one study using *Pseudomonas aeruginosa* PAO1, impedance decreased by approximately 22% after 24 h of biofilm growth in 1:10 TSB and approximately 25% in a 5% oil–water emulsion. The measurement frequency was optimized separately for the two media (1.26 kHz for 1:10 TSB and 200 kHz for the oil–water emulsion), showing that EIS response strongly depends on the sensor configuration and surrounding medium. These impedance changes were correlated with biofilm measurements obtained by confocal laser scanning microscopy and other biomass measurements [87,89].

#### EIS and Electrochemical Sensors in Meat/Poultry Contexts

Work from USDA-ARS and others has used EIS to characterize microbial growth and fouling on food-processing contact surfaces, demonstrating its sensitivity to changes in surface contamination under conditions relevant to meat/poultry plants (stainless-steel coupons and water–protein–fat matrices) [90,91,92]. More generally, electrochemical biosensors (amperometric, potentiometric, impedimetric) have been configured with antibodies or aptamers for food-spoilage and pathogen detection (e.g., *E. coli*, *Salmonella*), using changes in electron transfer or interfacial impedance upon cell binding to functionalized electrodes [90,91,92].

Food-processing-relevant studies further support the feasibility of EIS while illustrating the need for application-specific calibration. Yang and Reyes-De-Corcuera developed a continuous-flow system using *Pseudomonas putida* associated with chicken carcass material and monitored biofilm formation by EIS over bacterial suspension concentrations ranging from to CFU/mL. Changes in charge-transfer resistance and biofilm resistance were correlated with conventional plate counts. Although this study provides evidence under food-processing-relevant laboratory conditions, it was not conducted as a commercial plant validation study and therefore does not establish routine field performance [93].

Potential poultry and meat applications:

EIS and related impedimetric sensors could potentially be incorporated into processing-relevant locations such as chiller-water loops, scalding systems, conveyor drip areas, or sanitation/CIP circuits. These configurations represent proposed or prototype applications rather than demonstrated routine commercial deployments. Integrating electrodes into plant systems would require validation of sensor performance under variable temperature, ionic strength, protein and fat loading, sanitizer exposure, fouling, and cleaning cycles. Food-processing-relevant laboratory studies provide a basis for these applications, but commercial plant-scale validation remains necessary before such systems can be considered operational monitoring tools [87,89,90,91,92].

### 5.2. Quartz Crystal Microbalance (QCM): Principle for Attached Biomass

Quartz crystal microbalance (QCM) is a surface-sensitive technique that detects biofilm accumulation by measuring changes in the resonance frequency of a piezoelectric quartz crystal [94]. As cells and extracellular material attach to the electrode-coated surface, the added mass decreases the resonance frequency, which is proportional to the mass per unit area, as described by the Sauerbrey relation for thin, rigid films [23,95,96]. In liquid environments and for soft, viscoelastic biofilms, QCM is commonly operated in the dissipation mode (QCM-D), which simultaneously tracks frequency shifts and energy dissipation. This dual measurement captures not only total coupled mass (including bound water) but also changes in viscoelastic properties, enabling assessment of biofilm structure, mechanical behavior, and matrix remodeling during development [45,97]. For bacterial biofilms, cells and EPS attaching to the gold-coated quartz surface produce negative frequency shifts and changes in dissipation that track biomass accumulation, matrix stiffening/softening, and response to stressors. Detailed kinetic profiles can be obtained, including stable mass-coupled plateaus followed by changes in frequency and dissipation associated with biofilm reorganization or maturation [45,98].

#### QCM Applications Relevant to Meat and Poultry

Most published QCM studies have been conducted under laboratory conditions using model biofilms and stainless-steel or stainless-steel–like surfaces. Direct validation in commercial meat and poultry processing plants remains limited. Nevertheless, these studies provide a strong mechanistic basis for adapting QCM to monitor biofilm formation on processing equipment under plant-like conditions. QCM-D has been applied to monitor adsorption and assembly of biofilm matrix proteins and EPS analogs on stainless steel–like surfaces, enabling controlled studies of how surface treatments (e.g., protein-conditioning films from blood or meat exudates) influence biofilm adhesion and viscoelasticity [45,97]. Gold-coated QCM electrodes have been used to study how biofilm-forming bacteria respond to chemical stress (e.g., metals and disinfectants), with frequency and dissipation changes indicating biofilm stiffening or partial detachment, like sanitizer-induced effects on processing-line equipment [45,98].

In principle and in some prototype systems, QCM sensors can be mounted in flow cells fed with meat-processing rinse waters or brine/chiller water, providing continuous mass-based signals of biofilm accumulation on a sensor surface that mimics biofilm accumulation on stainless steel belts or pipe interiors [92,98]. Such QCM-based approaches offer high sensitivity to nanogram-level mass changes, but practical deployment in meat/poultry plants must address cleaning chemistry, mechanical robustness, and protein and fat fouling.

### 5.3. Surface Acoustic Wave (SAW): Principle for Attached Biomass

Surface acoustic wave (SAW) devices use interdigitated transducers on a piezoelectric surface to generate and detect acoustic waves that travel along the surface; changes in mass, viscoelastic films, or mechanical properties at the surface alter the wave’s velocity and attenuation. When a biofilm forms on the functionalized SAW surface, additional mass and viscoelastic coupling alter the acoustic wave, producing measurable changes in phase, velocity, and insertion loss at a fixed frequency, or shifts in resonant frequency [92,99].

Variants such as shear-horizontal SAW (SH-SAW) and Love-wave devices confine energy to a guiding layer near the surface, improving sensitivity in liquid environments such as processing water. Love-wave devices are highly sensitive SH-SAW sensors that utilize guiding layers (e.g., SiO_2_) on piezoelectric substrates to detect real-time changes in viscosity, mass, or biomolecules [100]. These devices are particularly effective in liquid environments due to their low noise, high-frequency stability, and the trapped energy within the guiding layer. The sensing surface is usually coated with metals (e.g., Au), oxides (ZnO, In_2_O_3_, graphene oxide), or polymers (polyethylenimine, polyethylene naphthalate) to capture target cells or improve biocompatibility and corrosion resistance [92].

#### SAW Sensors and (Bio)fouling in Food Systems

SAW biosensors have been extensively investigated to detect foodborne pathogens such as *E. coli* and *Salmonella*. In these systems, antibodies are immobilized along the acoustic propagation path, and bacterial binding produces measurable changes in the acoustic signal. Reported configurations include dual-channel SAW devices that enable reference-compensated measurements, langasite-based sensors, and microfluidically integrated shear-horizontal SAW (SH-SAW) chips capable of monitoring bacterial binding in real time at concentrations relevant to food safety [92,99].

A SAW biofilm sensor has also been demonstrated as a real-time microsystem capable of detecting biofilm growth and removal, with integrated electrodes that enable on-chip treatment using electric fields in combination with other modalities. In this configuration, acoustic measurements track biofilm accumulation on the surface by increases in acoustic attenuation and phase shift; treatment-induced removal yields a partial return toward baseline, analogous to what one would desire in online monitoring of cleaning efficacy on meat or poultry equipment [99].

For meat and poultry adaptation, some features are particularly relevant. For instance, the ability to operate wirelessly and at high frequency is attractive for monitoring inaccessible or hazardous zones in processing plants (e.g., inside ducts, humid chillers) [92]. SAW syringe filters and PEN-based SAW devices have been proposed as inline monitors of microbial load in food-related liquids [92]. Compatibility of SH-SAW and Love-wave geometries with aqueous and complex media could enable acoustic monitoring of attached biomass even in protein- and fat-rich environments if fouling is managed [92,99].

### 5.4. Current Status of Meat and Poultry Applications

USDA-ARS work on impedance characterization of microbial growth on processing equipment demonstrates that stainless-steel-based EIS sensors can detect biofilm contamination under realistic plant conditions, suggesting the feasibility of embedding such sensors in poultry processing lines for proactive sanitation monitoring [91]. SAW-based biosensors for foodborne pathogens, including Love-wave and SH-SAW devices targeting *E. coli*, highlight architectures (polymer guiding layers, microfluidic chips, syringe-filter formats) that can be repurposed to detect attached biomass and microbial pathogens in poultry chiller water, scald tanks, or carcass rinse streams [92,99]. Identification of biofilm “hot spots” in meat-processing environments that handle pork, poultry, and beef has revealed persistent colonization, typically on conveyor belts, drains, and equipment junctions [101], underscoring the need for online sensors in these locations. EIS electrodes or SAW/QCM devices bonded to surrogate coupons could provide continuous, early-warning biofilm indices in such hotspots [85].

**Table 5:** Comparison of Biosensors and Real-Time Platforms.

Platform	Information Content for Biofilms	Typical Implementation Format for Meat/Poultry Contexts	Plant-Relevant Strengths	Key Limitations and Challenges	Current Maturity for Meat/Poultry Plants	Evidence Base
Electrochemical impedance spectroscopy (EIS) and related impedimetric sensors [93]	Sensitive to attached biomass at the electrode surface; can track biofilm formation and removal in real time and can provide semi-quantitative estimates of biofilm load when calibrated against reference methods.	Interdigitated or planar stainless steel/gold electrodes integrated into flow cells, water loops, or contact surfaces such as chiller water, scalders, or conveyor drips; some platforms may be functionalized with antibodies or aptamers for target organisms such as *Salmonella* or *Campylobacter*.	Compatible with wet, protein- and fat-rich matrices; suited to online or inline monitoring; relatively fast measurements; can be coupled with sanitation validation rigs and digital monitoring systems.	Requires protection against heavy fouling and sanitizer residues; signals are influenced by temperature, ionic strength, and soil composition; interpretation usually requires calibration against biomass or microbiological endpoints for a given application.	Promising candidate technology for plant-adjacent and pilot-scale monitoring; requires further plant-scale validation, ruggedization, and application-specific calibration before routine commercial use.	Plant-like laboratory studies; limited food-processing validation
Quartz crystal microbalance (QCM and QCM-D) [77,82]	Provides highly sensitive measurement of attached mass per unit area, with QCM-D offering added insight into whether the film is relatively rigid or soft and hydrated.	Usually implemented as bench-top or pilot-scale crystal sensors in controlled flow cells; can be coupled to microfluidic systems carrying meat- or poultry-relevant isolates and soils for sensor development and mechanistic studies	Highly sensitive to early adhesion and subtle mass changes; useful for mechanistic study of growth, EPS accumulation, and sanitizer-induced detachment under controlled conditions	Sensor surfaces are delicate, difficult to protect in harsh plant environments, and sensitive to temperature and hydrodynamic instability; integration into large, heavily soiled equipment areas is challenging.	Best suited at present for laboratory and pilot-loop applications rather than routine inline deployment in commercial meat and poultry plants.	Plant-like laboratory systems; limited food-processing validation
Surface acoustic wave (SAW) and related acoustic devices such as SH-SAW and Love-wave [81,87]	Detects attached biomass in a label-free manner and, in some device designs, can provide information on interfacial loading behavior associated with developing biofilms.	Commonly built as microfabricated chips embedded in flow cells or cartridges and sometimes combined with biorecognition layers for microbial fouling or pathogen detection in aqueous food-related systems.	High sensitivity, miniaturization potential, multiplexing potential, and compatibility with real-time measurement in compact modules.	Performance can be disrupted by temperature variation, viscosity changes, and heavy soil; protective packaging must withstand cleaning chemistries and mechanical stress; engineering for CIP compatibility remains a major hurdle	Currently at proof-of-concept to early pilot stage for biofilm and microbial sensing, with additional engineering needed before robust use in poultry or meat processing lines.	Laboratory proof-of-concept; conceptual adaptation for meat and poultry processing

EIS should currently be regarded as a promising candidate technology for real-time biofilm monitoring in meat and poultry processing rather than as a technology approaching routine commercial deployment. Laboratory and food-processing-relevant studies show that impedance measurements can track biofilm development and response to treatment, but the optimal frequency, electrode configuration, and signal response depend strongly on the organism, medium, surface, and operating conditions. Further validation under commercial plant conditions is needed to establish robustness in the presence of variable temperature, ionic strength, protein and fat residues, sanitizer residues, fouling, and multispecies biofilms. QCM remains primarily a laboratory and pilot-scale validation tool, while SAW-based approaches remain at the proof-of-concept to early-pilot stage for meat and poultry applications [85,93].

## 6. Microfluidic and High-Throughput Platforms for Biofilm Detection Method Development

These platforms are increasingly central to biofilm research in meat/poultry processing, enabling mechanistic studies under realistic conditions, rapid disinfectant screening, and the acceleration of new detection methods. When coupled with strains of bacteria isolated from processing plants, these systems allow investigators to examine biofilm behavior under plant-relevant shear, temperature, and sanitizer-pulse regimes, quantify relationships among biomass, viability, EPS, and cleaning, and benchmark novel sensors and assays in a controlled yet scalable format [102,103,104].

### 6.1. How These Platforms Support Sensor and Method Development

Microfluidic and robotic high-throughput platforms provide a versatile testbed for rapid, comparative evaluation of new biofilm detection concepts before field deployment. For example, emerging electrochemical impedance or amperometric sensors can be interfaced with microfluidic flow cells carrying biofilms formed from poultry plant isolates, enabling controlled assessment of sensor response as a function of biofilm thickness, EPS content, and sanitizer exposure. Similarly, optical or fluorescence-based detection schemes (e.g., specific EPS probes, ATP-based assays) can be benchmarked against established endpoints such as CFU, crystal violet biomass, and EPS-component assays across the same isolate collection [103,105].

A key advantage is the ability to decouple biofilm properties from background environmental noise. By varying flow, temperature, and sanitizer pulse profiles in microfluidic systems, researchers can systematically test how experimental conditions affect sensor signals and define operating envelopes in which a given method remains robust. At the same time, robotic screening facilitates head-to-head comparisons of multiple detection strategies on the same panel of broiler-plant isolates, yielding empirical data on sensitivity, specificity, throughput, and cost per assay. This integration of microfluidic realism with high-throughput automation accelerates the translation of laboratory-proven biofilm metrics into deployable, plant-relevant tools for real-time monitoring of biofilm burden and disinfectant efficacy [102,103,106].

### 6.2. Microfluidic Models of Meat/Poultry-Plant Conditions

Microfluidic and millifluidic devices provide plant-like laboratory systems for developing and validating biofilm detection technologies under controlled processing conditions [106]. Flow cell and microchannel systems with defined shear profiles allow researchers to track biofilm attachment, structural development and validation, and detachment under controlled hydrodynamic conditions, clarifying how shear influences early adhesion, aggregation, and the formation of streamer-like structures in process water. Parallel channel designs can also be operated at 4–12 °C using cold-adapted poultry plant isolates (e.g., *Pseudomonas, Aeromonas, Acinetobacter*), enabling direct comparison of EPS composition, metabolic activity, and sanitizer tolerance between low- and high-temperature biofilms [102,103].

When coupled with automated valves or dosing modules, these platforms can replicate the “pulse-and-rest” sanitizer exposures (e.g., chlorine, peracetic acid, quaternary ammonium compounds) that equipment surfaces experience during cleaning and disinfection. Real-time optical or electrochemical readouts in lab-on-a-chip systems support mechanistic studies of sanitizer kinetics, EPS-mediated diffusion barriers, and spatially heterogeneous cell killing, helping explain why some biofilms persist despite industry-standard disinfectant concentrations. Collectively, these systems provide a tunable, high-resolution framework for hypothesis-driven studies that link plant-scale operating conditions to biofilm physiology and resistance mechanisms [102,103,106,107]. Although these platforms are primarily laboratory tools, they provide an important bridge between mechanistic biofilm studies and subsequent validation of detection methods under commercial meat and poultry processing conditions.

### 6.3. Robotic High-Throughput Biofilm Assays

Robotic high-throughput platforms accelerate the evaluation of biofilm detection methods and sanitation strategies by enabling standardized testing of large numbers of isolates under identical experimental conditions. In one recent broiler processing plant study, hundreds of isolates (including *Pseudomonas, Aeromonas, Escherichia, Citrobacter*, and others) were grown in microtiter-based biofilm assays, then exposed to commercial disinfectants at recommended concentrations, exposure times, and temperatures using automated dispensing and incubation. This approach demonstrated that while disinfectants can substantially reduce planktonic loads and mono-species biofilms, they often fail to fully eradicate biofilms, with survival rates ranging from about 7% to over 50% depending on the disinfectant and strain [103].

High-throughput readouts can be tailored to multiple biofilm metrics that directly relate to cleaning performance. For instance, absorbance-based crystal violet assays quantify total attached biomass [108]. ATP bioluminescence assays indicate metabolic activity/viability [109]; and BacTiter Glo-type assays [103] measurements are related to cellular ATP and metabolic activity. Fluorescence or enzymatic assays quantify EPS components (e.g., proteins, polysaccharides, eDNA) [27]. By correlating these metrics across a matrix of disinfectant type, concentration, contact time, and biofilm age, robotic platforms generate multivariate data that link specific biofilm phenotypes (thick EPS-rich, dispersed, multilayered) to resilience under realistic cleaning and disinfection regimens [103]. Such datasets are especially valuable for risk-based prioritization of disinfectant–strain–surface combinations most likely to yield persistent biofilms in poultry plants.

Current evidence indicates that microfluidic and robotic platforms are best regarded as laboratory and pilot-scale tools for developing, optimizing, and validating biofilm detection technologies rather than as methods intended for routine use in commercial meat and poultry processing plants. Their principal value lies in providing standardized, plant-like conditions that accelerate the translation of emerging microbiological and sensor-based approaches into practical monitoring tools (Table 6).

## 7. Translating Emerging Biofilm Detection Technologies to Commercial Meat and Poultry Processing

The successful implementation of emerging microbiological and sensor-based biofilm detection technologies depends on their ability to operate reliably under commercial meat and poultry processing conditions, integrate with existing sanitation verification programs, and demonstrate clear regulatory and economic value [7,110,111,112,113].

### 7.1. Sampling and Validation in Plants

Emerging sensor platforms and microbiological assays need validation against biofilms formed under typical wet, cold, protein- and fat-rich conditions on stainless steel, plastics, and elastomers in commercial poultry plants. Chemical residues from sanitation (quats, peracetic acid, chlorinated compounds) can quench optical signals, foul electrodes, and inhibit downstream PCR, so protocols must explicitly test interference and define acceptable windows after sanitation [7,110,111,113].

Pilot-scale trials in commercial or pilot plants should compare sensor outputs with culture-based enumeration, qPCR/amplicon sequencing, and surface imaging on matched sites across several weeks and sanitation cycles. Meaningful performance metrics for implementation include limit of detection on rough, soiled surfaces; false-positive and false-negative rates under real soil conditions; time to an actionable result within a shift; and robustness to temperature fluctuations across chillers, evisceration, and cut-up areas [7,110,111,112,113].

Networked sensor nodes mounted on drains, hard-to-clean structural members, and CIP loops can provide continuous or high-frequency signals for biofilm risk rather than episodic snapshots. These nodes can include impedance or electrochemical probes, optical fibers, or inline turbidity/fluorescence sensors embedded in CIP return lines to detect changes in organic load or sloughed biofilm during cleaning [110,112,113].

To be useful, sensor data must be aggregated into plant-level dashboards with clear traffic-light indicators tied to specific zones and equipment, plus anomaly-detection models that flag deviations from each line’s historical baseline. Rules or machine-learning-based decision engines can then link signal thresholds to actions such as targeted re-cleaning of a belt, intensified sanitation of a CIP circuit, or triggered microbiological confirmation sampling, all while logging events for verification and trend analysis [110,112,113].

### 7.2. Coupling with Robotics and Plant Operations

Fixed sensors can be integrated with mobile systems, such as collaborative robots or guided sprayers, to close the loop between detection and response in hygiene niches that are ergonomically difficult for workers to access. For example, a high-risk signal could trigger a programmed robotic inspection or a directed foam application, reducing worker exposure to chemicals while improving consistency in biofilm control [110,112,113].

Digital records from these automated responses, including timestamps, locations, sanitizer type, and confirmation test results, can be integrated into the establishment’s food safety management system and used to document verification of sanitation preventive controls. Over time, linking microbiological outcomes (e.g., *Salmonella* and *Listeria* results) to sensor and robot-action histories will help refine trigger thresholds and identify persistent harborage sites [7,110,112,113].

### 7.3. Regulatory and Economic Considerations

For broad adoption, any emerging biofilm-detection tool must be positioned as a verification or monitoring aid that complements, rather than replaces, required FSIS-compliant environmental sampling and validation activities. FSIS directives already acknowledge biofilms as a contributor to persistent *Listeria monocytogenes* contamination, so plants can frame sensor systems as enhancements to existing sanitary design, pre-operational inspection, and corrective action programs [108].

From an economic perspective, the business case hinges on preventing high-cost events such as product holds, recalls, and brand damage, as well as reducing labor-intensive, non-targeted re-cleaning. Cost–benefit analyses should incorporate equipment and integration costs, maintenance and calibration, incremental sanitation chemical or water use, and potential reductions in line downtime, environmental positives, and insurance or liability risks.

Successful implementation will depend on technologies that provide rapid, interpretable information on biofilm risk, withstand the harsh physical and chemical conditions of meat and poultry processing plants, integrate with digital sanitation management systems, and complement existing FSIS verification programs. Many of the technologies reviewed remain at the laboratory or pilot scale. Established fluorescence-based screening and molecular assays have greater practical applicability, whereas EIS and other real-time sensor platforms require additional validation under commercial meat and poultry processing conditions before routine implementation can be recommended.

## 8. Conclusions

Biofilm detection in meat and poultry processing environments is best approached as a tiered process in which rapid screening, confirmatory testing, and investigative or validation methods serve different purposes. Routine plant screening is currently best supported by established approaches such as visual inspection, ATP bioluminescence, protein- and carbohydrate-based swabs, and validated fluorescence-based surface screening. These methods can rapidly identify sites requiring corrective action or additional sampling but generally do not, by themselves, establish the presence, identity, or viability of a biofilm-forming microorganism.

Confirmatory testing is best accomplished using culture-based methods and validated targeted molecular assays such as qPCR; dPCR can provide absolute quantification and may be useful for low-abundance targets or assay validation, although its application remains more specialized. Molecular results require appropriate interpretation because DNA persistence, extraction efficiency, gene-copy-number variation, PCR inhibition, and cellular physiological state can affect results. PMA/PMAxx-qPCR and RNA-based assays can provide complementary information regarding membrane integrity or transcriptional activity but should not be interpreted as definitive measures of viability or culturability.

Advanced microscopy, sequencing, EPS-focused analyses, and controlled-flow or microfluidic systems are primarily investigative and validation tools. They are valuable for characterizing biofilm architecture, community composition, matrix components, persistence, sanitation response, and the mechanisms underlying contamination that routine screening cannot resolve. Their principal role is therefore to support root-cause investigations, validate sanitation interventions, and evaluate emerging monitoring technologies rather than replace routine plant monitoring.

Real-time sensor platforms, including EIS, QCM, and SAW, remain emerging technologies for meat and poultry processing. EIS has demonstrated promising biofilm-monitoring capability in laboratory and food-processing-relevant systems, but optimal sensor configurations and responses remain dependent on the organism, matrix, electrode configuration, and operating conditions. QCM and SAW similarly provide valuable opportunities for real-time or near-real-time monitoring but require additional validation under commercial processing conditions. Proposed applications involving chiller loops, scalders, conveyor drip areas, and CIP systems should therefore be regarded as conceptual or prototype applications unless demonstrated in operating facilities.

Overall, the most practical strategy is not to replace existing sanitation verification programs with a single advanced technology, but to combine rapid screening with appropriately selected confirmatory methods and targeted investigative tools. This tiered approach allows processors to obtain rapid operational information while retaining culture and molecular methods for confirmation and advanced imaging, sequencing, EPS analyses, and sensor platforms for validation, root-cause investigation, and future development. Continued validation under the wet, cold, protein- and fat-rich, chemically variable conditions of commercial meat and poultry processing facilities will determine which emerging technologies can ultimately transition into routine monitoring.

## Figures and Tables

**Table 1:** Selected U.S. poultry-associated foodborne illness outbreaks, 2017–2026. These examples are illustrative rather than exhaustive. Only outbreaks linked to poultry foods/products are included; outbreaks caused solely by contact with live backyard poultry are excluded from this table.

Period	Poultry-Associated Food Vehicle	Implicated Organism	Illnesses/Hospitalizations/Deaths	Primary Reference
2018	Prepackaged ready-to-eat chicken salad (Triple T Specialty Meats/Fareway)	*Salmonella* Typhimurium	265 cases; 94 hospitalized; 1 death; 8 states	[9]
2017–2019	Raw turkey products; outbreak strain also detected in raw turkey pet food and live turkeys	*Salmonella* Reading	356 cases; 132 hospitalized; 1 death; 42 states + DC	[10]
2018–2019	Raw chicken products; outbreak strain also detected in raw chicken pet food and live chickens	*Salmonella* Infantis	129 cases; 25 hospitalized; 1 death; 32 states	[11]
2020–2021	Ground turkey	*Salmonella* Hadar	33 cases; 4 hospitalized; 0 deaths; 14 states	[12]
2021	Raw frozen breaded stuffed chicken products	*Salmonella* Enteritidis	36 cases; 12 hospitalized; 0 deaths; 11 states	[13]
2021	Frozen fully cooked chicken products supplied by Tyson Foods	*Listeria monocytogenes*	3 cases; 3 hospitalized; 1 death; 2 states	[13]
2021–2025	Ready-to-eat meat and poultry products distributed by Yu Shang Food	*Listeria monocytogenes*	24 cases; 22 hospitalized; 8 pregnancy-associated illnesses; infant deaths reported; 9 states	[14]
2024–2026	Prepared refrigerated meals, including chicken fettuccine Alfredo; later investigation included other pasta-containing meals	*Listeria monocytogenes*	28 cases; 27 hospitalized; 7 deaths; 19 states; 1 pregnancy-associated fetal loss	[14]

**Table 2:** Tiered framework for screening, confirmation, and investigation of biofilms in meat and poultry processing environments.

Tier	Purpose	Typical Methods/Signals	What the Result Means	Appropriate Confirmation/Follow-Up
Tier 1—Rapid screening	Rapidly identify areas requiring attention and prioritize sanitation or additional sampling	Visual inspection; ATP bioluminescence; protein/carbohydrate swabs; fluorescence imaging (e.g., Bactiscan/Biofinder); other rapid surface assays	Indicates organic residue, elevated ATP/metabolic signal, fluorescence, or other evidence of contamination. Does not by itself establish a biofilm or identify the organism.	Culture and/or targeted molecular testing (qPCR) of a matched environmental sample, with appropriate sampling controls
Tier 2—Confirmatory testing	Determine whether a suspected site contains microorganisms and/or a persistent biofilm-associated population	Culture; targeted qPCR; dPCR where increased analytical sensitivity or absolute quantification is required; microscopy with appropriate stains	Establishes microbial presence and, where appropriately designed, identifies or quantifies a target organism. Interpret molecular detection with consideration of DNA persistence, extraction efficiency, inhibition, and viability limitations.	Culture confirmation, sequencing, or microscopy depending on the question and target; repeat sampling can establish persistence
Tier 3—Investigative/validation	Determine biofilm structure, composition, persistence, mechanism, or response to sanitation; validate emerging sensors	CLSM/epifluorescence; SEM; OCT; amplicon sequencing/metagenomics; EPS analyses; EIS; QCM; SAW; microfluidic/controlled-flow systems	Provides mechanistic, spatial, community-level, or sensor-performance information. Many methods are laboratory or pilot-scale and are not intended as stand-alone routine plant tests.	Compare with established microbiological endpoints (culture and/or appropriately validated molecular assays), biomass measurements, microscopy, and sanitation-performance data

**Table 6:** Characteristics, evidence base, technology readiness, and applications of microfluidic and high-throughput platforms for the development and validation of biofilm detection methods in meat and poultry processing environments.

Platform	Primary Purpose	Evidence Base	Technology Readiness	Contribution to Biofilm Detection
Microfluidics [17,94]	Plant-like laboratory model	Plant-like laboratory	Research	Sensor development; validation
Lab-on-a-chip [15,90]	Integrated sensing	Laboratory	Emerging	Rapid assay development
Robotic HTS [91,95]	Large-scale screening	Laboratory; poultry isolates	Validation	Benchmark detection methods

## Data Availability

No new data were created or analyzed in this study. Data sharing is not applicable to this article.
